# Structure Conservation and Differential Expression of Farnesyl Diphosphate Synthase Genes in Euphorbiaceous Plants

**DOI:** 10.3390/ijms160922402

**Published:** 2015-09-15

**Authors:** Dong Guo, Hui-Liang Li, Shi-Qing Peng

**Affiliations:** Key Laboratory of Tropical Crop Biotechnology, Ministry of Agriculture, Institute of Tropical Bioscience and Biotechnology, Chinese Academy of Tropical Agricultural Sciences, Haikou 571101, China; E-Mails: guodong@itbb.org.cn (D.G.); lihuiliang@itbb.org.cn (H.-L.L.)

**Keywords:** farnesyl diphosphate synthase, Euphorbiaceae, gene expression, development

## Abstract

Farnesyl diphosphate synthase (FPS) is a key enzyme of isoprenoids biosynthesis. However, knowledge of the *FPSs* of euphorbiaceous species is limited. In this study, ten *FPSs* were identified in four euphorbiaceous plants. These *FPSs* exhibited similar exon/intron structure. The deduced FPS proteins showed close identities and exhibited the typical structure of plant *FPS*. The members of the *FPS* family exhibit tissue expression patterns that vary among several euphorbiaceous plant species under normal growth conditions. The expression profiles reveal spatial and temporal variations in the expression of *FPSs* of different tissues from Euphorbiaceous plants. Our results revealed wide conservation of *FPSs* and diverse expression in euphorbiaceous plants during growth and development.

## 1. Introduction

Euphorbiaceae is one of the largest plant families and consists of more than 7000 species. Euphorbiaceus species are evolutionally-diversified, carry distinct physiologies, and have complex traits adapting to dynamic environmental conditions [1]. There are many economically-important plants in Euphorbiaceae, such as the rubber tree (*Hevea brasiliensis*), the cassava (*Manihot esculenta*), and the castor bean (*Ricinus communis*). The rubber tree is the most widely cultivated species for commercial production of natural rubber (*cis*-polyisoprene) for tires and other products [2]. The cassava is a tropical crop that stores important quantities of starch in its roots. The high starch content makes cassava a desirable energy source both for human consumption and industrial biofuel applications [3]. The castor bean is cultivated in the tropical and subtropical areas of the world for oil production and as an ornamental plant [4].

Isoprenoids constitute a versatile class of compounds fulfilling major physiological functions [5]. The isoprenoid pathway constitutes the most diverse and widespread metabolic pathway of all prokaryotes and eukaryotes, resulting in the biosynthesis of a large number of primary as well as secondary metabolites [6]. In plants isoprenoids are formed by the mevalonate (MVA) pathway in the cytosol [7,8] and the 1-deoxy-d-xylulose 5-phosphate (DXP)/2-Cmethyl-d-erythritol 4-phosphate (MEP) pathway in plastids [9,10]. The MVA pathway is primarily responsible for the synthesis of sesquiterpenes, triterpenes including brassinosteroids, larger molecules such as dolichols, and even macromolecular polyisoprene (natural rubber) [6,11,12]. Farnesyl diphosphate synthase (FPS) is a key enzyme in isoprenoids biosynthesis, which catalyzes the consecutive condensations of dimethylallyl diphosphate (DMAPP) or geranyl diphosphate (GDP) with isopentenyl pyrophosphate (IPP) to produce farnesyl diphosphate (FDP) [8]. FDP serves as a precursor for sesquiterpenoids, sterols, brassinosteroids, triterpenoids, polyprenols, side chains of ubiquinone, and polyisoprenoids such as natural rubber [13,14]. However, little is known of the *FPS* genes in the Euphorbiaceus species. In this study, the gene structure, phylogenetic characteristics, and expression patterns of Euphorbiaceae plants *FPSs* were identified and described. Our results revealed wide conservation of FPSs and diverse expression profiles in Euphorbiaceous plants during growth and development.

## 2. Results

### 2.1. Cloning, Identification and Structure Analysis of the Euphorbiaceous Plants FPSs 

**Figure 1:**
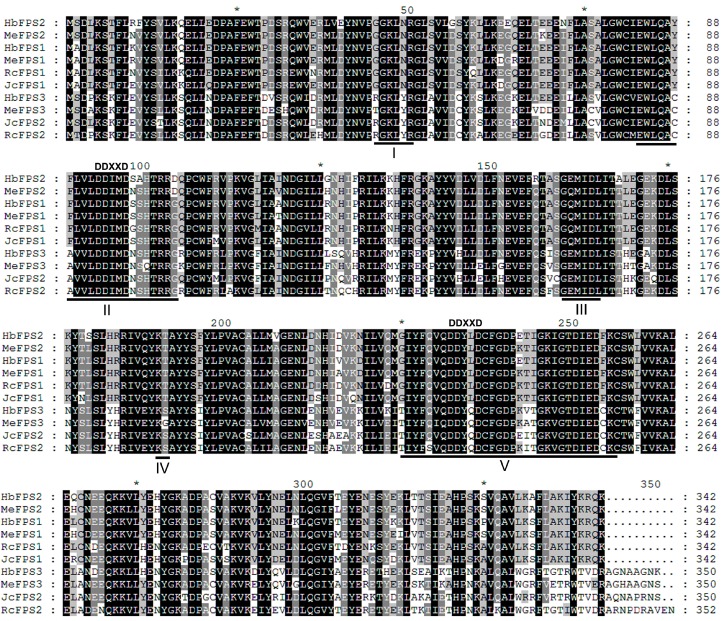
Amino acid sequence alignment of FPSs from four euphorbiaceus species. Identical and conserved amino acid residues are denoted by black and gray backgrounds, respectively. The five conserved domains of prenyltransferases are underlined and numbered. The highly-conserved aspartate-rich motifs (DDXXD) is present in domains II and V.

To identify the potential members of the *FPS* family in euphorbiaceous plants, we used *Arabidopsis FPSs* (*AtFPS1* and *AtFPS2*) as queries and obtained all possible *FPSs* by searching the genome database of the rubber tree (*Hevea brasiliensis*), cassava (*Manihot esculenta*), castor bean (*Ricinus communis*), and Jatropha (*Jatropha curcas*). Three members in the rubber tree (designated as *HbFPS1*, *HbFPS2*, and *HbFPS3*), three members in the cassava (designated as *MeFPS1*, *MeFPS2*, and *MeFPS3*), two members in the castor bean (designated as *RcFPS1*, *RcFPS2*), and two members in the *Jatropha* (designated as *JcFPS1*, *JcFPS2*) were identified on the basis of the BLASTP search. The full-length cDNAs of the ten *FPSs* were PCR amplified, cloned and sequenced. The deduced proteins of the FPSs ranged from 342 to 352 amino acids (predicted molecular mass = 39.37 to 40.80 kDa) with isoelectricpoints ranging from 4.85 to 6.06 (Table 1). The deduced FPS proteins contained the five conserved regions identified by Chen *et al.* [15] that are characteristic of prenyltransferases that synthesize isoprenoid diphosphates with E-double bonds (Figure 1). The highly conserved aspartate-rich motif DDXXD was present in domains II and V. Ten FPSs identified from euphorbiaceous plants showed more than 65.2% amino acid identity and the maximum percentage of amino acid sequence identities was found between HbFPS1 and MeFPS1 (95.61%, respectively) (Table 2).

**Table 1:** Basic information of ten *FPSs* identified from four euphorbiaceous plants

Gene	GenBank Accession No.	Gene Size (bp)	ORF (bp)	Predicted Protein		
				Size (aa)	*M*_W_ (kDa)	pI
*HbFPS1*	Z49786	4690	1029	342	39.41	5.94
*HbFPS2*	KT306000	4171	1029	342	39.55	5.07
*HbFPS3*	KT306001	3710	1053	350	40.27	6.06
*MeFPS1*	KT306002	4349	1029	342	39.48	5.68
*MeFPS2*	KT306003	5666	1029	342	39.57	5.86
*MeFPS3*	KT306004	4296	1053	350	40.13	5.18
*RcFPS1*	KT306005	5720	1029	342	39.37	5.30
*RcFPS2*	XM_002522756	3583	1059	352	40.73	4.85
*JcFPS1*	XM_012219426	3977	1029	342	39.43	5.30
*JcFPS2*	XM_012215689	3886	1053	350	40.80	5.72

**Table 2:** The percentage of FPS amino acid identity in four euphorbiaceous plants.

	HbFPS2	HbFPS3	MeFPS1	MeFPS2	MeFPS3	RcFPS1	RcFPS2	JcFPS1	JcFPS2	EpFPS
HbFPS1	90.94	68.71	95.61	94.44	67.84	91.81	69.30	93.27	66.37	89.47
HbFPS2		65.20	90.64	91.81	65.20	86.84	66.08	87.43	63.45	84.50
HbFPS3			68.13	69.88	87.14	68.42	84.29	67.84	84.86	67.84
MeFPS1				94.74	67.84	90.64	68.71	92.98	64.91	89.77
MeFPS2					69.30	90.94	68.71	91.81	66.96	89.47
MeFPS3						66.96	81.71	66.08	84.00	66.08
RcFPS1							67.54	91.52	65.79	89.18
RcFPS2								67.25	82.57	66.67
JcFPS1									66.37	90.64
JcFPS2										64.91

### 2.2. Phylogenetic Analysis

**Figure 2:**
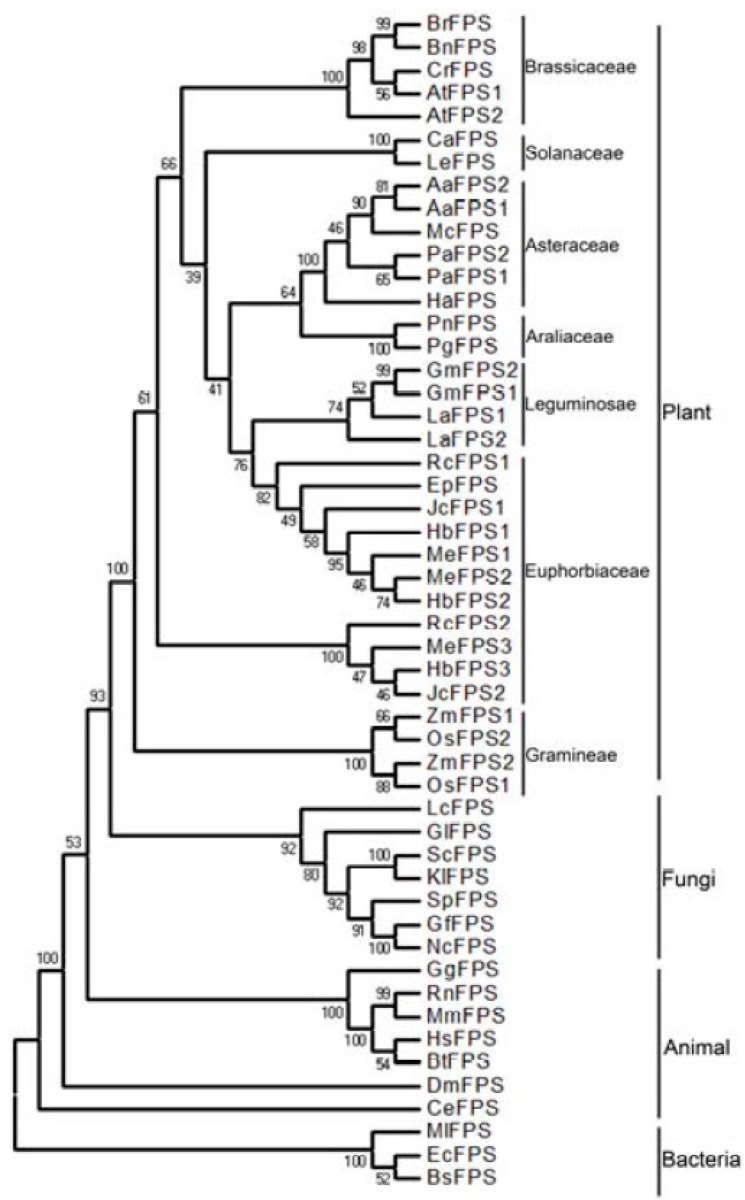
Phylogenetic tree of FPSs from different organisms constructed by the neighbor-joining method on MEGA. The accession numbers of FPS known proteins in GenBank are listed as follows: BrFPS, XP_009128999; BnFPS, CDY68039, CrFPS, XP_006281527; AtFPS1, AAB49290; AtFPS2, AAB07248; Ca, CAA59170; LeFPS, AAC73051; AaFPS1, AAC49452; AaFPS2, AAD17204; McFPS, ABS11699; PaFPS1, CAA57892; PaFPS2, CAA57893; HaFPS, AAC78557;PnFPS, AAY53905; PgFPS, AAY87903;GmFPS1, ACU21393; GmFPS2, XP_003534984;LaFPS1,AAA86687; LaFPS2, AAA87729; EpFPS, ACN63187; ZmFPS1, AAQ14871; ZmFPS2, ACG34051; OsFPS1, BAA19856; OsFPS2,AAU43998; LcFPS, BAD15361; GlFPS,ACB37020; ScFPS, P08524; KlFPS,CAA53614; OSpFPS,14230;GfFPS, Q92235;NcFPS, Q92250; GgFPS, P08836; RnFPS, P05369; MmFPS, AAl09445; HsFPS, NP_001995; BtFPS, AAL58886; DmFPS, CAA08919; CeFPS, CAB03221; MlFPS, BAA25265; EcFPS, BAA00599; BsFPS, Q08291.

Phylogenetic and molecular evolutionary analyses were conducted using MEGA version 6 [16] by comparing ten FPS from euphorbiaceous plants with known FPS sequence from a wide range of different organisms including bacteria, fungi, plants, and animals (Figure 2). The results indicated that ten FPSs from euphorbiaceous species appeared at the base of the clade of the plant kingdom, and that FPSs evolved from a common ancestor. Moreover, FPSs from euphorbiaceous plants were clustered into two distinct subgroups. One subgroup contained HbFPS1, HbFPS2, MeFPS1, MeFPS2, RcFPS1, EpFPS, and JcFPS1, which was more closely related to the FPS of legume plants. The other subgroup contained HbFPS3, MeFPS3, RcFPS2, and JcFPS2.

### 2.3. Intron and Exon Organization of FPSs

**Figure 3:**
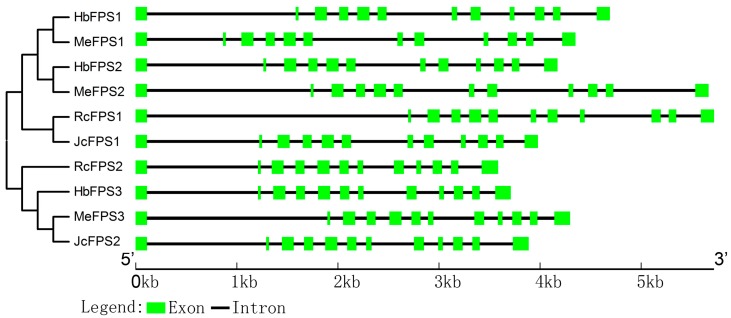
Neighbor-joining phylogenetic tree and intron-exon structures. The phylogenetic tree (part of the left side) was constructed from FPSs using the MEGA 6.0 program with the NJ method. Intron and exon structural organization of *FPS* genes are described on the right side. Introns and exons are represented by black lines and colored boxes, respectively.

We analyzed the intron and exon structure of ten *FPSs* from the rubber tree, the cassava, the castor bean, and the *Jatropha* (Figure 3). All these *FPSs* contained twelve exons and eleven introns. Although introns differ in length, these introns were typically flanked by GT and AG boundaries.

### 2.4. Structure Prediction and Homology Modeling of the FPSs 

**Figure 4:**
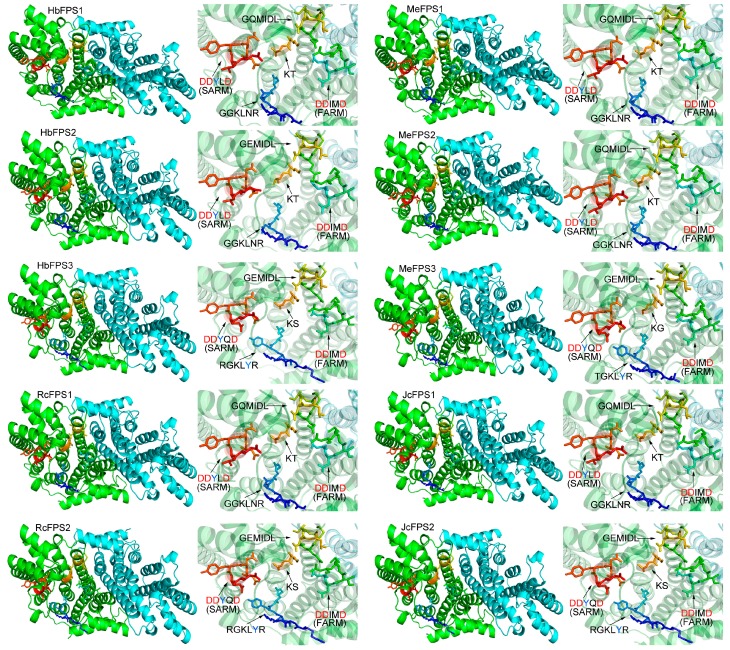
Representation of the predicted 3D structure model and the active sites of the FPSs from euphorbiaceus species. The graphics at the right side are the close-up views of the active sites. Motif-II (First Asp-rich motif, FARM), Motif-III, motif-IV and Motif-V (Second Asp-rich motif, SARM) are shown in sticks.

In order to obtain a reasonable theoretical structure of the euphorbiaceous plant FPSs, protein homology modeling was performed using a Swiss model server. To predict the 3D structure of the FPSs, a 3D structure at 2.20 Å of *Artemisia Spiciformis* FPS1 (PDB id: 4kk2.1) was used as a template, which shares 80.59%, 75.29%, 66.07%, 79.71%, 79.41%, 66.57%, 80.00%, 66.27%, 79.71% and 65.36% sequence identity with HbFPS1-3, MeFPS1-3, RcFPS1-2, and JCFPS1-2, respectively. The predicted 3D model of FPSs was validated with the QMEAN server [17] for model quality estimation. The total QMEAN-score (estimated model reliability between 0 and 1) of the predicted 3D models for the ten FPSs are 0.796 (Z-score: −1.34), 0.773 (Z-score: −2.02), 0.778 (Z-score: −1.86), 0.798 (Z-score: −1.28), 0.805 (Z-score: −1.07), 0.758 (Z-score: −2.43), 0.800 (Z-score: −1.22), 0.772 (Z-score: −2.04), 0.797 (Z-score: −1.30) and 0.771 (Z-score: −2.07), respectively. It indicates that all the sequences of FPSs match the homologous templates well on the server, so the models are reliable. The overall predicted structures of FPSs with substrate are similar to the template 4kk2.1. The five conserved motifs are shown in sticks. Motif-II (First Asp-rich motif, FARM), Motif-III, motif-IV and Motif-V (Second Asp-rich motif, SARM) within the FPSs have the similar orientation in the predicted 3D structure (Figure 4). The Asn residue in motif-I of HbFPS1-2, MeFPS1-2, RcFPS1, and JcFPS1 have the similar predicted 3D structure; also, the similar predicted 3D structure is found in the Tyr residue in motif-I of HbFPS3, MeFPS3, RcFPS2, and JcFPS2. However, the Tyr, instead of ASn, residue forms a different predicted 3D structure, where Asn residue forms an open structure, the Tyr residue forms a cyclic structure.

### 2.5. Expression Analysis of FPSs in Euphorbiaceous Plants Tissues

**Figure 5:**
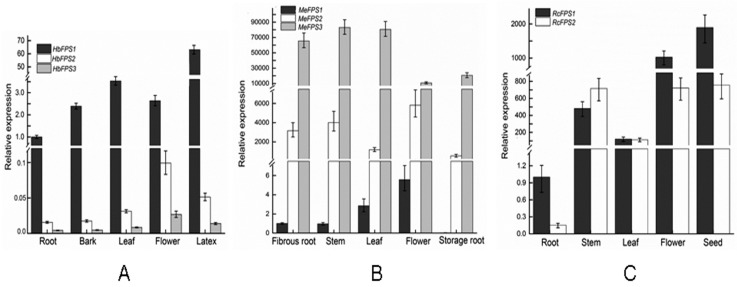
The expression of the *FPSs* from euphorbiaceus species. The amount of *FPS* mRNA was normalized by *ACT* mRNA in the rubber tree and in the cassava, 18S RNA gene in the castor bean. Each value is the mean ± SE of three biological replicates (*n* = 3). (**A**) Rubber tree; (**B**) Cassava; and (**C**) Castor bean.

In order to characterize the expression profile of FPS in euphorbiaceous plants, we analyzed the tissue-specific expression pattern of *FPSs* in three euphorbiaceous species. In the rubber tree, *HbFPS1* was predominant in the latex, revealed more than a 20-fold difference in the expression levels of different organs. *HbFPS2* and *HbFPS3* had similar expression profiles, *HbFPS2* and *HbFPS3* were expressed in all the tested tissues at different levels, with the highest transcription occurring in flowers, followed by latex, barks, leaves, and root. *HbFPS1* showed more than 30-fold higher levels of transcript abundance than *HbFPS2* and *HbFPS3* in different organs (Figure 5A). We also compared the transcripts of *FPSs* in each tissue in the cassava and found that the expression levels of *MeFPS1*, *MeFPS2*, and *MeFPS3* had similar expression profiles, but *MeFPS3* revealed more than a 100-fold difference in the expression levels than *MeFPS1* and *MeFPS2* in different organs (Figure 5B). In the castor bean, *RcFPS1* and *RcFPS2* were expressed in all the tested tissues at different levels, with the highest transcription occurring in seeds, followed by flowers, stems, leaves, and root (Figure 5C)

## 3. Discussion

Plants contain small farnesyl diphosphate synthase isozyme families. cDNAs encoding *FPS* have been cloned and characterized from various plant species [18,19,20,21,22,23,24,25,26]. Arabidopsis contains two genes, *FPS1* and *FPS2*, encoding three FPS isozymes: FPS1L, FPS1S and FPS2. The *FPS1* encodes FPS1S and FPS1L, which differ only by an N-terminal extension of 41 amino acid residues that targets FPS1L into mitochondria [19,20], whereas the *FPS2* encodes *FPS2* that shares 90.6% amino acid identity with FPS1 isozymes [21]. Three FPS isoforms have also been discovered in both maize and *Artemisia tridentate* [22,23]. In humans, only a single *FPS* encodes for FPS. Due to the alternative splicing in the first exon of human *FPS*, multiple splice variants are generated which encode two FPS isoforms: a shorter cytoplasmic/peroxisomal form, and a longer isoform which is a mitochondrial targeting peptide [24]. Although one *FPS* (*HbFPS1*) from the rubber tree and one *FPS* (*EpFPS*) from the *Euphorbia* had been characterized [25,26], knowledge of the FPS genes of euphorbiaceous plants is limited. In this study, ten *FPSs* were identified in Euphorbiaceous species, including three members in the rubber tree, three members in the cassava, two members in the castor bean, and two members in the *Jatropha.* Sequence and phylogenetic analysis results showed wide conservation of *FPSs* in euphorbiaceous plants.

The members of the *FPS* family exhibit tissue expression patterns that vary among several plant species. In Arabidopsis, *FPSs* are expressed in all organs throughout plant development, albeit at greatly different levels. *FPS1* is widely expressed in all tissues throughout plant development, whereas expression of *FPS2* is mainly concentrated in floral organs, seeds, and the early stages of seedling development [27,28]. In *Ginkgo biloba*, *GbFPS* had high transcription in roots and leaves, and low in stems [29], reflecting the fact that the biosynthesis of ginkgolides and bilobalide occurs in roots and leaves [30]. In *Euphorbia pekinensis*, the highest *EpFPS* expression level was detected in roots, in which terpenoids are synthesized [26]. In the rubber tree, *HbFPS1* is expressed predominantly in the laticifers and is likely to encode the enzyme involved in natural rubber biosynthesis [25]. The expression of *HbFPS2* and *HbFPS3* is not cell-type specific. *HbFPS2* and *HbFPS3* are possibly involved in isoprenoid biosynthesis of a housekeeping nature. Our results revealed that all of the eight *FPS* genes were differentially expressed in all tissues tested either in their transcript abundance or expression patterns under normal growth conditions. 

Our results showed that a substantial number of *FPSs* which were previously identified and characterized in well studied model plants are conserved in important Euphorbiaceous plants. Despite broad conservation across the euphorbiaceous species, these *FPSs* also exhibited diverse expression patterns.

## 4. Experimental Section

### 4.1. Plant Materials and Treatments

Rubber tree (*Hevea brasiliensis* cultivar RRIM 600), castor bean (*Ricinus communis* cultivar A202), and cassava (*Manihot esculenta* cultivar SC8) obtained from Institute of Tropical Bioscience and Biotechnology, were planted in the experimental farm of the Chinese Academy of Tropical Agricultural Sciences in Hainan Island in China (20°N, 110°E). Fresh leaves, flowers, roots, fruits, and barks were immediately ground to form powder in liquid nitrogen and stored at −70 °C or immediately used to extract nucleic acid. The latex of rubber tree was allowed to drop directly into liquid nitrogen in an ice kettle. The frozen latex powder was then stored at −70 °C or used immediately to extract RNA. 

### 4.2. Cloning and Identification of FPS Genes 

Total RNA was extracted from the rubber tree latex [31] and from other tissues [32]. cDNA was synthesized by reversely transcribing 1 μg total RNA using a PrimeScript™ RT-PCR kit (Takara, Dalian, China) according to the manufacturer’s instructions. To identify the *FPS* homologs in *H. brasiliensis*, we used *Arabidopsis FPS* genes (*AtFPS1* and *AtFPF2*) as queries and BLAST analysis of genome database of rubber tree (DDBJ/EMBL/GenBank under the accession: GenBank: AJJZ01000000), cassava (http://www.phytozome.net/cassava) [33], castor bean (http://castorbean.jcvi.org) [4], and jatropha *(*http://www.kazusa.or.jp/jatropha/) [34]. The contigs of putative *FPS* genes were then assembled. The cDNA of putative *FPSs* were amplified by primers based on the assembled sequences (Table 3). The primers were designed using the Primer Generator (http://www.med.jhu.edu/medcenter/primer/primer.cgi). The PCR products were cloned in the pMD19-T cloning vector (TaKaRa, Dalian, China) and sequenced. The sequence was performed using the ABI BigDye^®^ Terminator Sequencing Kits in ABI3700 DNA sequencer. Afterward, their sequences were analyzed in GenBank by using the BLAST program. The isoelectric point (pI) of FPS was predicted using the compute pI/*M*_W_ software (http://www.expasy.ch/tools/pi_tool.html). The percentage of FPS amino acid identity in four euphorbiaceous plants were done with Clustal W2 (http://www.ebi.ac.uk /Tools/msa/clustalw2/). The gene structure schematic of *FPSs* identified from four euphorbiaceous plants was drawn using the web server GSDS (http://gsds.cbi.pku.edu.cn/). Multiple amino acid sequence alignment and phylogenetic tree analysis were performed using the MEGA 6.0 software.

**Table 3:** Gene specific primers of *FPSs* used for RT-PCR amplification.

Gene	Forward (5'→3')	Reverse (5'→3')
*HbFPS1*	TCCATGGCGGATCTGAAGTCAACT	CATCCAGTCTTTGTCCATGTATCTG
*HbFPS2*	AATCCATGTCTGATCTGAAGTCGA	ATCCAATCTTTGTCCATGTTCTTG
*HbFPS3*	ATGAGCGATCCAAAATCCAAGTTCTTGG	ATGTTAATCCTCAGCTCATTTTAGAGT
*MeFPS1*	CTCTGTTTTCAGTTTTTCTCCCCAATCT	CAATCTTTATCCATGTATCTGGATA
*MeFPS2*	CACTCTTCATTCACTCG AATCTCCG	CATATTAAGTGTTTACTTAAATAATAA
*MeFPS3*	GATATGAGCCAGTAAAGTTCCACAGTT	TTCTGAACCATTAGAAGAACAAGAAC
*RcFPS1*	AGCTTCATTCATTCTTTTCTCTCC	GATGATAAAAACCATTCATTCAATT
*RcFPS2*	GATTCAGAATTGTTCTTCAAAAGCGC	GAATCACAAAGTTGACAAGGAACCC
*JcFPS1*	TCAATCTCTCCTCACTACTGCCCTCC	CGCATTATTCGGCATCATCCAATCAT
*JcFPS2*	GCCCTTTCATATCGAACGGTAATAACAT	AAGTTTCATTTCCCATTCTAATGTTC

### 4.3. Homology Modeling and Structure Prediction 

Protein sequences of ten FPS were submitted to the Swiss-Model server (http://swissmodel.expasy.org) [35] to perform sequence analysis, and *Artemisia Spiciformis* farnesyl diphosphate synthase 1 (PDB id: 4kk2.1) was applied as a template. The catalytically- and enzymatically-important residues of FPSs were displayed using the Pymol software (Delino Scientific, San Carlos, CA, USA).

### 4.4. Expression Analysis

Quantitative real-time RT-PCR (qPCR) was conducted using the primers presented in Table 4. The primers were designed using the Beacon Designer (http://www.premierbiosoft.com). qPCR was performed using the fluorescent dye SYBR-Green (Takara, Dalian, China) and the BIO-RAD CFX96 qPCR system (Bio-Rad, Hercules, CA, USA). The reactions were carried out as follows: 30 s at 95 °C for denaturation, 5 s at 94 °C, 20 s at 60 °C, and 20 s at 72 °C for amplification. Three biological replicates were carried out and triplicate quantitative assays for each replicate were performed. A rubber tree actin gene [36], a cassava actin gene [37], and a castor bean 18S RNA gene [38] were amplified as an internal control. The relative abundance of transcripts was calculated according to the Bio-Rad CFX Manager (Version1.5.534) of BIO-RAD CFX96.

**Table 4:** Primers for *FPSs* used for qRT-PCR amplification.

Gene	Forward (5'→3')	Reverse (5'→3')
*HbFPS1*	TGAAAGCTATAAGAAACTAGTAACCTCT	TCATCCAGTCTTTGTCCATGTATC
*HbBFPS2*	GAACGAAAGCTATGAGAAACTAACC	TCATCCAATCTTTGTCCATGTTCT
*HbFPS3*	GGAACCAGATGGACAGTTGATAG	ACTAGGCAAATGCTGGTAATAGG
*HbACT*	CACCACCAGAGAGAAAGTACAG	GATGGACCAGACTCATCGTATTC
*MeFPS1*	GAAAGCTATGAGATATTAGTGACT	ATCATCATCATTCAATCTTTATCCA
*MeFPS2*	AAAGCTATGAGAAACTAGTAACCT	CCCTGTTTTTATTTATTTCTGTCT
*MeFPS3*	AACCAGATGGACAGTTGAGAGAG	AAGAACAAGAACCAAAGCAGATG
*MeACT*	CAGTGGTCGACAACTGGTAT	ATCCTCCAATCCAGACACTGT
*RcFPS1*	AGTGTTGAAGTCTTTCCTGGC	CTAGCATTATTCGCACGATCC
*RcFPS2*	GCTTTGTGGGGAAGATTTACAG	ACAAAGTTGACAAGGAACCCAA
*Rc18S RNA*	TTGGTGGAGCGATTTGTC	CCCAGAACATCTAAGGGCAT

## 5. Conclusions

In conclusion, ten *FPSs* were cloned from four euphorbiaceus species. All ten *FPSs* exhibited similar exon/intron structure. All FPSs contains contained the five conserved regions. All of the *FPS* genes were differentially expressed in all tissues tested either in their transcript abundance or expression patterns under normal growth conditions. The expression profiles reveal spatial and temporal variations in the expression of *FPS* genes of different tissues from three Euphorbiaceous plants.
